# Green fluorometric strategy for simultaneous determination of the antihypertensive drug telmisartan (A tentative therapeutic for COVID-19) with Nebivolol in human plasma

**DOI:** 10.1038/s41598-023-30400-w

**Published:** 2023-03-02

**Authors:** Mohamed M. Salim, Aya saad Radwan, Ghada M. Hadad, Fathalla Belal, Mahmoud M. Elkhoudary

**Affiliations:** 1 Department of Pharmaceutical Analytical Chemistry, Faculty of Pharmacy, Mansoura University, Mansoura, Egypt; 2 Department of Pharmaceutical Chemistry, Faculty of Pharmacy, Horus University- Egypt, New Damietta, Egypt; 3 Department of Pharmaceutical Analytical Chemistry, Faculty of Pharmacy, Suez Canal University, Ismailia, Egypt

**Keywords:** Analytical chemistry, Green chemistry

## Abstract

Telmisartan (TEL) and Nebivolol (NEB) are frequently co-formulated in a single dosage form that is frequently prescribed for the treatment of hypertension, moreover, telmisartan is currently proposed to be used to treat COVID19-induced lung inflammation. Green rapid, simple, and sensitive synchronous spectrofluorimetric techniques for simultaneous estimation of TEL and NEB in their co-formulated pharmaceutical preparations and human plasma were developed and validated. Synchronous fluorescence intensity at 335 nm was used for TEL determination (Method I). For the mixture, the first derivative synchronous peak amplitudes (D^1^) at 296.3 and 320.5 nm were used for simultaneous estimation of NEB and TEL, respectively (Method II). The calibration plots were rectilinear over the concentration ranges of 30–550 ng/mL, and 50–800 ng/mL for NEB and TEL, respectively. The high sensitivity of the developed methods allowed for their analysis in human plasma samples. NEB`s Quantum yield was estimated by applying the single-point method. The greenness of the proposed approaches was evaluated using the Eco-scale, National Environmental Method Index (NEMI), and Green Analytical Procedure Index (GAPI) methods.

## Introduction

Coronavirus disease 2019 (COVID-19), the extremely contagious viral disease produced by the severe acute respiratory syndrome coronavirus 2 (SARS-CoV-2), has had a devastating impact on the demography of the world, causing more than 6 million deaths globally as of March 2022, making it the greatest significant global health emergency since the influenza pandemic of 1918^1^.

The World Health Organization (WHO) had to proclaim SARS-CoV-2 a global pandemic on March 11, 2020, after the first instances of this primarily respiratory viral infection were first detected in Wuhan, Hubei Province, China, in late December 2019. This was due to the virus's rapid global spread. Since it was deemed a global pandemic, COVID-19 has wreaked havoc on numerous nations and overrun numerous healthcare systems^2^.

Several works of literature have been developed to predict the number of COVID-19 cases all over the world, such as in Egypt^3^, Saudi Arabia^4^ and Russia, and Brazil^5^.

Cardiovascular diseases (CVDs) are the leading cause of death worldwide. At the turn of the twentieth century, Less than 10% of deaths globally occurred from CVDs, but by 2001, that number had risen to 30%. Around 80% of all CVD deaths worldwide occur in low- and middle-income nations^6^.

**Figure 1:**
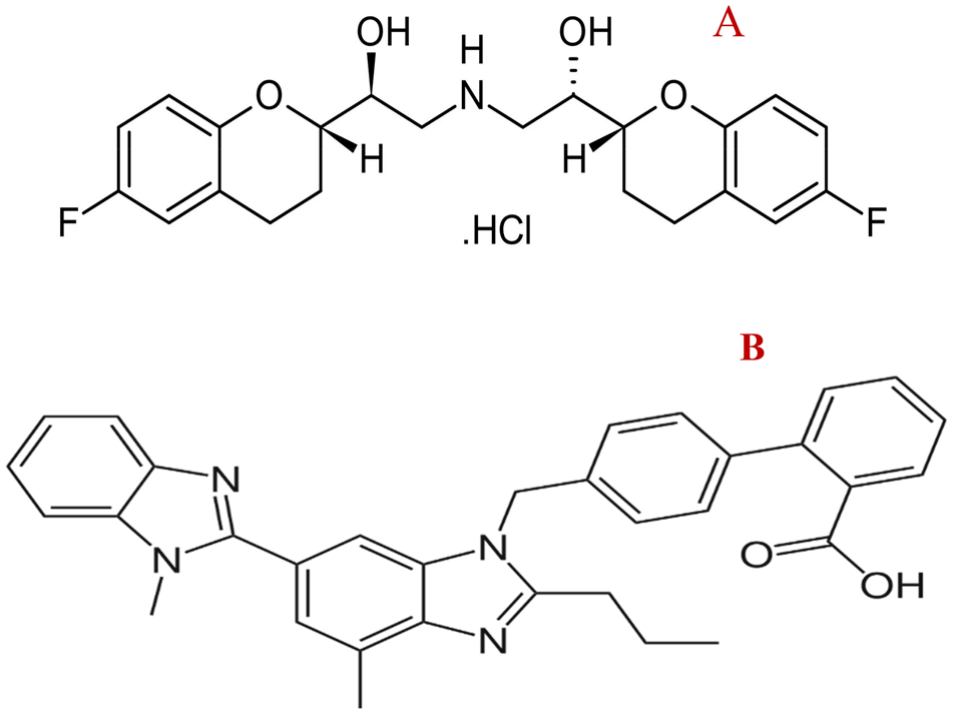
Structural Formulae of (**A**) Nebivolol HCl, (**B**) Telmisartan.

Nebivolol hydrochloride, NEB, (Fig. 1A) is Butanedioic acid;1-[4-(2-methoxyethyl) phenoxy]-3-(propan-2-ylamino) propan-2-ol^7^. It is a widely prescribed beta1-selective adrenergic receptor blocker used for the treatment of hypertension, chronic heart failure, cardiac arrhythmia, angina pectoris, acute myocardial infarction, and migraine prophylaxis ^8^. The analysis of NEB in different matrices has been reported using a wide range of analytical techniques, viz UV spectrophotometry^9,10^, spectrofluorimetry^11,12^, HPLC^13–15^, HPTLC^16^, and electrochemistry^17^.

Telmisartan, TEL, (Fig. 1B) is 2-[4-Methyl-6-(1-methylbenzimidazol-2-yl) 2propylbenzimidazol-1-yl] methyl] phenyl] benzoic acid^7^. It is a frequently administered angiotensin II receptor blocker type 1, which protects the renal and vascular system against any harm caused by CVDs^18^. Interestingly, TEL was recently proposed as a promising treatment for COVID-19 by the same mechanism it works for CVDs^19,20^. Moreover, the clinical trials demonstrate that the angiotensin II receptor blocker (ARB) drug; Telmisartan has a significant role in the mortality rate reduction among hospitalized patients with COVID-19 and those in the intensive care unit (ICU)^21^.

Spectrophotometry^22,23^, spectrofluorimetry^24^, HPLC^22,25,26^, HPTLC^27^and UPLC^28^ techniques were utilized for the estimation of TEL in its pharmaceutical preparations and biological fluids.

The literature survey showed a UV spectrophotometric method^29^ as well as HPLC methods^30,31^ for simultaneous estimation of NEB and TEL in their co-formulated tablets.

Up to date, no spectrofluorometric method has been yet reported for the concurrent quantitative analysis of both drugs. Therefore, there is still a need for a method that is simple, more sensitive, and selective for quantitative analysis of this binary mixture to be utilized in their simultaneous analysis of dosage forms and biological fluids.

The sensitivity, simplicity and selectivity of the outstanding spectrofluorometric technique are merits that overweighed the reported spectrophotometric and HPLC methods^32,33^. Constant-wavelength, variety angle, or constant-energy scanning modes of synchronous fluorescence spectroscopy (SFS) are all modes that can be selected for developing spectral simplification and selectivity^34,35^. Constant wavelength monochromator scanning mode, the one that maintains the difference between excitation and emission wavelengths constant (Δλ), is widely utilized to analyze combined pharmaceutical preparations and human plasma. Moreover, using SFS along with derivative amplitude helps to obtain an add-on selectivity^36^.

Significant overlapping was found in the emission spectra of both NEB and TEL and, hence, conventional spectrofluorimetry could not be applied to assess the two drugs simultaneously. Therefore, we resorted to synchronous spectrofluorimetry to solve this problem.

Moreover, the discussed greenness of the SFS method added further attributes to the developed method and was proven by applying each of GAPI^37^, Eco. Scale and NEMI^38^. Green chemistry first appeared in the early 2000s, with the development of the concept of the green analytical chemistry (GAC) approach^39,40^. GAC is endorsed by the majority of analysts because it aims to improve the environment and health by eliminating or reducing the use of harmful chemicals during analysis^41^. Due to the health and environmental risks associated with solvents commonly used in SFS, green SFS methods have attracted interest from the analytical community with the goal of replacing environmentally unfriendly analytical methods with cleaner ones.

A greener SFS process is implemented by minimizing solvent consumption and using less toxic and environmentally friendly alternatives to the hazardous and toxic organic solvents; and recycling solutions via scaling up to large-scale preparative analytical techniques^41–43^.

Therefore, this work aimed to develop and validate a rapid, simple, environmentally benign, and sensitive synchronous spectrofluorimetric technique for the determination of TEL alone (Method I) and a first derivative (D^1^) SFS method for the concurrent estimation of TEL and NEB (Method II) in their co-formulated dosage forms and spiked human plasma samples.

## Experimental

### Apparatus

Cary Eclipse Spectrofluorometer (Agilent Technologies, Inc., USA) equipped with Xenon flash lamp was used for recording the fluorescence spectra. The slit widths of both emission and excitation monochromators were kept at 20 nm. The data was stored and then manipulated via Cary Eclipse Scan Application Software version: 1.2 (147) (Agilent Technologies, Inc., USA). D^1^ spectra were measured at 1 nm intervals and the filter size was 20.

Vortex Mixer: Gemmy Industrial Corp. (Taiwan) Model: IVM-300 p.

Centrifuge Model 2-16P (Germany).pH-meter; model Consort, Belgium.

### Materials and Reagents

- Nebivolol HCl (NEB, 99.4% purity), was kindly supplied by Sigma Company for Pharmaceutical Industries, Quesna, Egypt.
- Telmisartan (TEL,99.91% purity) was gently given by International Drug Agency for Pharmaceutical Industry (IDI), Cairo, Egypt
- **Pharmaceutical Preparations**
- Nevilob Tablets (Batch No. 2033518) labeled to include 5 mg NEB per tablet, a product of Marcyrl Pharmaceutical Industries, Cairo, Egypt.
- Micardis Tablets (Batch No. 906949) are labeled to include 40 mg TEL per tablet, a product of Boehringer Ingelheim Pharma, Germany.
- Both preparations were purchased from a local Pharmacy in the Egyptian market.
- Methanol, acetonitrile, ethanol, Phenol, hexane, Tween-80, 99% cetrimide, 95% sodium dodecyl sulphate, methylcellulose, and β-cyclodextrin were bought from Sigma-Aldrich, Germany. All surfactants were prepared in distilled water at a concentration of 1% w/v or v/v.
- Also, 96%acetic acid, sodium acetate trihydrate, sodium hydroxide, and boric acid were bought from the same source.
- Acetate buffer (0.2 M) was prepared using sodium acetate trihydrate and acetic acid and its pH was adjusted at 3.7 − 5.5, while borate buffer (0.2 M) was composed of boric acid and potassium chloride, and pH was adjusted to cover the range of (6–9) using sodium hydroxide.
- Human plasma samples were obtained from Blood Bank, Mansoura University Hospital (Mansoura, Egypt), and kept frozen at − 20 °C until use after gentle thawing.

### Standard solutions

#### Stock standard solutions

10.0 mg of either NEB or TEL were accurately weighed and dissolved separately in methanol in a 100-mL volumetric flask to prepare a stock standard solution (100.0 μg/mL). Aliquots of these stock solutions were further diluted with distilled water to obtain standard solutions containing (5.0 μg/mL). The standard solutions were found to be stable for 7 days when kept in the refrigerator at 4 °C.

#### Working standard solutions

Aliquots of NEB and TEL were accurately transferred from standard solutions (5.0 μg/mL) into a series of 10-mL volumetric flasks, then 1-mL of acetate buffer of pH 4 was added, completed to the mark with distilled water to prepare solutions covering the working concentration range (30–550 ng/mL) and (50–800 ng/mL) for NEB and TEL, respectively.

#### Determination of quantum yield of NEB

- *Standard solution of NEB*

10.0 mg of NEB was accurately weighed and transferred into a 100-mL volumetric flask and dissolved in methanol to prepare a stock standard solution (100.0 μg/mL).

- * Standard solution of phenol*

25.0 mg of phenol was accurately weighed and dissolved in hexane in a 25- mL volumetric flask to prepare a stock standard solution (1000.0 μg/mL). An aliquot of this stock solution was further diluted with the same solvent to obtain a standard solution containing 100.0 μg/mL**.**

### Procedures

#### Construction of calibration graphs

Aliquots of the standard solutions containing (30–550 ng/mL) for NEB and (50–800 ng/mL) for TEL were transferred into 10-mL volumetric flasks;1-mL aliquots of acetate buffer of pH 4 were added and then completed to the mark with distilled water. SFS Measurements of NEB and TEL were recorded at Δλ = 15, using 5 nm slit width and 600 nm/min as scanning rate. Store the recorded typical synchronized spectra using Cary Eclipse software with a filter size of 20 and a 1 nm interval to calculate the magnitude of the first derivative fluorescence spectra. Relative synchronous fluorescence intensities of TEL were recorded at 335 nm (method I), while 296.3 and 320.5 nm were the points at which the peak amplitudes of the first derivative spectra of NEB and TEL were recorded, respectively; (method II).

The calibration curves were obtained by plotting the peak amplitudes against their corresponding concentrations, thereafter the corresponding regression equations were derived.

#### Analysis of synthetic mixtures

Aliquots from NEB and TEL stock solutions were transferred into a 10 ml measuring flask, diluted with 1-mL aliquots of acetate buffer of pH 4 then completed to the mark with distilled water to reach final concentrations covering the medicinally-recommended ratio (1:8) for NEB/TEL, respectively. Then the procedure under “Construction of Calibration Graphs.” was applied. The percentage recoveries were then calculated using the Calibration Graphs or the corresponding regression equation.

#### Analysis of pharmaceutical preparations

Into 100-mL volumetric flask, a weighed amount of prepared tablets containing 5 mg of NEB, 40 mg of TEL and commenly used excepients were transferred, then 70 mL of methanol were added and the mixture was sonicated for 20 min. The volume was completed to the mark with the same solvent and then filtered. The filtrate was diluted with distilled water to obtain 5 μg/mL working solution. The procedures described under “Construction of Calibration Graphs” were then performed. The nominal content of the tablets was obtained using the corresponding regression equation.

#### Procedure for spiked human plasma

Transfer 0.5 mL aliquots of human plasma into a series of small centrifugation tubes then spike with suitable aliquots of TEL or NEB stock solutions. Acetonitrile was added up to 2 mL, to denature the plasma proteins. Vortex the samples for 30 s, followed by centrifuging for 20 min at 3600 rpm. The supernatants were aspirated carefully and filtered using 0.45 µm syringe filter. One mL of supernatant was transferred into a 10-mL measuring flask followed by 0.2 mL of acetate buffer of pH 4, and 0.8-mL of distilled water to give final concentration ranges of (50–150 ng/mL for TEL) (Method I) and (50–150 ng/mL for TEL) and (50- 200 ng/ml for NEB) (Method II).

#### Fluorescence quantum yield of NEB

Accurately, transfer 0.35 mL and 0.7 mL from the methanolic standard solutions of NEB (test solution) and phenol solution (indicator solution) into two 10-mL volumetric flasks. Three absorbance scans were performed for both the indicator and the test solutions, and the average absorbance values for each compound were calculated. Triplicate measurements of the emission spectra of both solutions of indicator and test were recorded at λ_ex_ 280.0 nm. The values of the integration peaks were calculated from the corrected fluorescence spectra.

## Results and discussion

**Figure 2:**
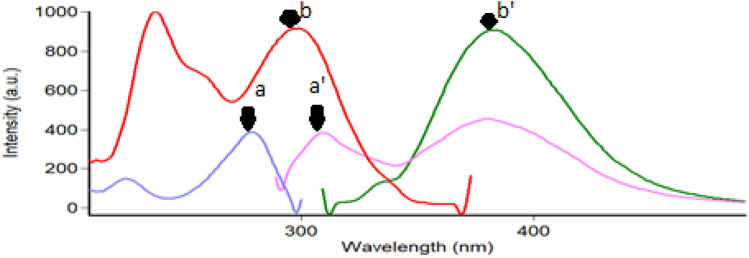
Excitation and emission spectra of NEB and TEL in acetate buffer of pH 4, where: (a, a´) : NEB(100.0 ng /mL) excitation and emission spectra.(b, b´) : TEL (30.0 ng/mL) excitation and emission spectra.

A strong native fluorescence of aqueous acetate buffer solution of pH 4 of NEB and TEL was observed at 300/382 nm following the excitation at 280/309 nm, respectively. As illustrated in (Fig. 2), significant overlapping in the emission spectra of the targeted compounds was observed. Hence, it was challenging to estimate such a combination in pharmaceuticals and biological matrices. The resolution of such a mixture was attempted by studying the SFS at different Δ λ settings. Two criteria were considered while developing the proposed method, firstly, we aimed to find a suitable wavelength where either of the drugs is only detectable. Secondly, the higher spectral contribution of TEL in the synchronous spectra of the mixture is because of its higher medical ratio in their combined dosage form (8:1), meanwhile, it has a more intense fluorescence signal than NEB.

### Optimization of experimental conditions

The various parameters that may alter the spectra of each NEB and TEL were studied as follows:

#### Choice of optimum Δ λ

**Figure 3:**
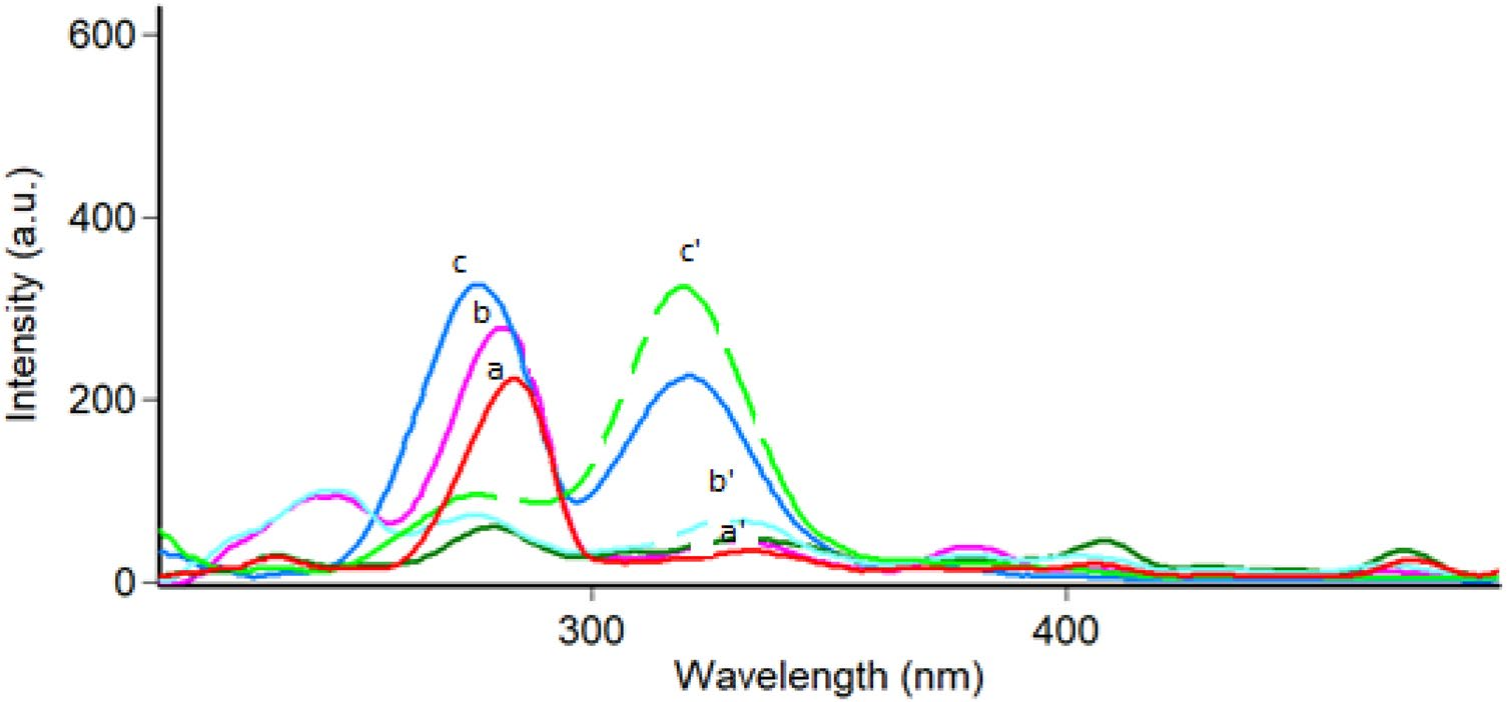
Effect of Δ λ on the relative synchronous fluorescence intensity of NEB (100.0 ng/ mL) and TEL (30.0 ng/ mL). where: (a, b, c) for Δ λ (15, 20, 40 nm), respectively for NEB (—) (a’, b’, c’) for Δ λ (15, 20, 40 nm), respectively for TEL (---).

**Figure 4:**
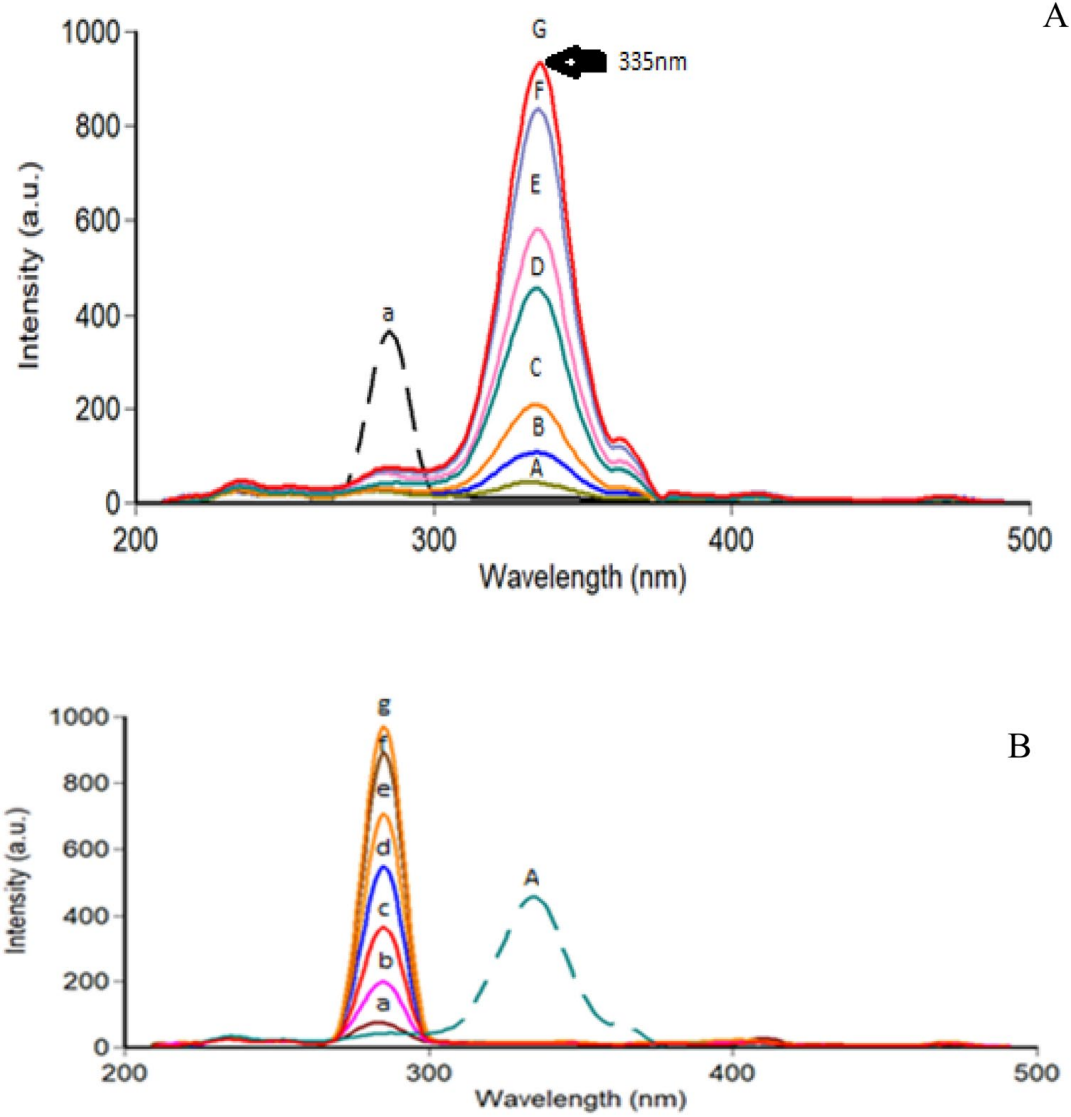
(**A**): Synchronous fluorescence spectra of different concentrations of TEL with a constant concentration of NEB. Where: (A -F): TEL at concentrations of (50.0, 100.0, 200.0, 400.0, 500.0,700.0 and 800.0 ng /mL).(a): NEB concentration (200.0 ng /mL). (**B**): Synchronous fluorescence spectra of different concentrations of NEB with a constant concentration of TEL. Where: (A -F): NEB at concentrations of (30.0, 100.0, 200.0, 300, 400.0,500.0 and 550.0 ng /mL). (a): TEL concentration (400.0 ng /mL).

**Figure 5:**
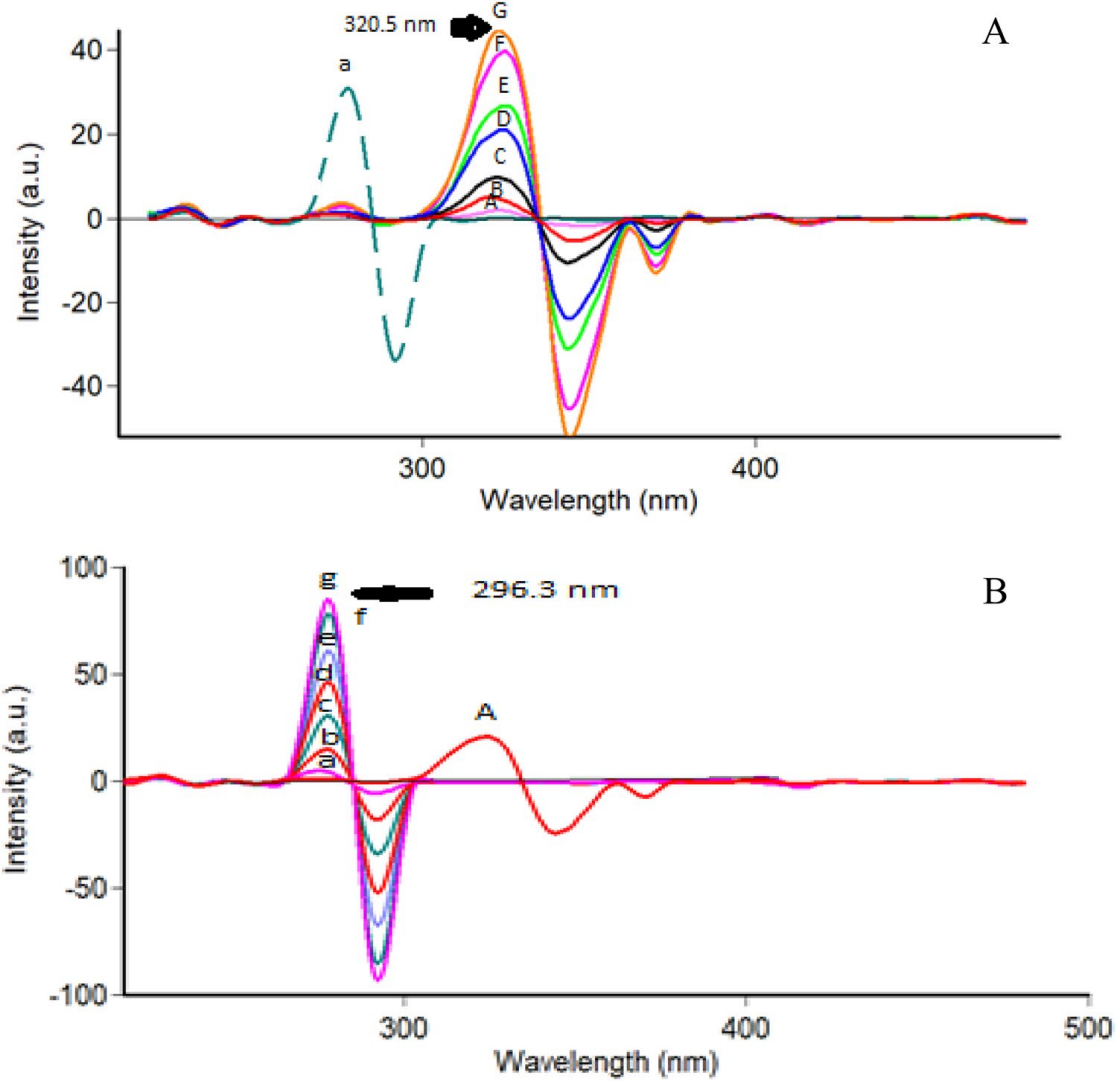
(**A**): First derivative synchronous fluorescence spectra of various concentrations of TEL with a constant concentration of NEB. Where: (A -F): TEL at concentrations of (50.0, 100.0, 200.0, 400.0, 500.0, 700.0 and 800.0 ng /mL). (a): NEB concentration (200.0 ng /mL). (**B**): First derivative synchronous fluorescence spectra of various concentrations of NEB with a constant concentration of TEL. Where:(a-f): NEB at concentrations of (30, 100, 200, 300, 400,500 and 550 ng /mL). (A): TEL concentration (400.0 ng /mL).

In a trial to reach ideal spectra, a wide range of Δ λ (15–120 nm) was tested (Fig. 3). It was observed that, the best Δ λ that matched the present analysis criteria were15 nm, 20 nm, 40 nm, and 60 nm, they gave distinct spectral shapes for both drugs. A compromise was done using Δ λ of 15 nm as it decreases the RSFI of TEL while keeping the relative synchronous fluorescence intensity of NEB still high.TEL could be measured without interference from NEB at 335 nm, yet a significant overlapping was still shown between the two SFS in the NEB region (Fig. 4). Consequently, the first derivative SFS technique has resorted to more selective detection of both NEB and TEL at zero crossing points at 296.3 nm and 320.5 nm; respectively (Fig. 5).

#### Effect of diluting solvent

**Figure 6:**
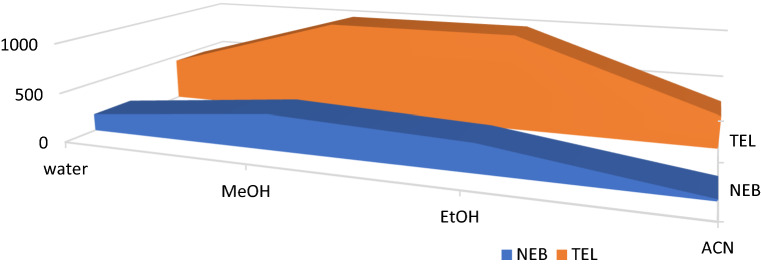
Effect of diluting solvents on the relative synchronous fluorescence intensity of (100.0 ng/ mL) of each NEB and TEL.

Solvents with different polarity index values were tested for their outcome on SF intensity using water, methanol, ethanol, and acetonitrile. No significant increase was noticed in SF intensity of NEB using water, methanol, ethanol, or acetonitrile. On the other hand, methanol, ethanol, and acetonitrile showed an increased SF intensity for TEL. Based on the formerly mentioned analysis criteria water was selected as the solvent of choice for both drugs as it decreases TEL's SF intensity, as illustrated in (Fig. 6) adding one more merit regarding the methods’ greenness.

#### Effect of pH

**Figure 7:**
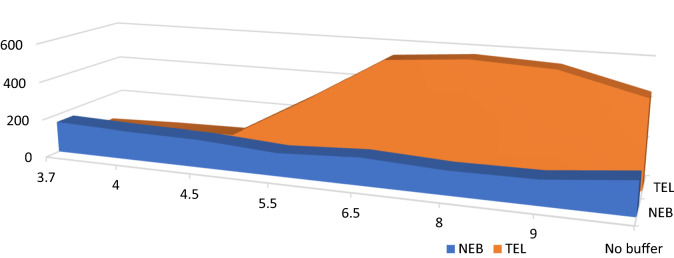
Effect of pH on the relative synchronous fluorescence intensity of (100.0 ng/ mL) for each NEB and TEL.

A wide pH range using 0.2 M of each of acetate (3.7–5.5) and borate buffer (6.0–9.0) was tested. 0.2 M acetate buffer (pH 4) decreased the SF intensity of TEL, while maintaining the SF intensity of NEB (Fig. 7) that’s why it was preferred to keep the pH adjusted at 4.

#### Effect of volume of buffer

**Figure 8:**
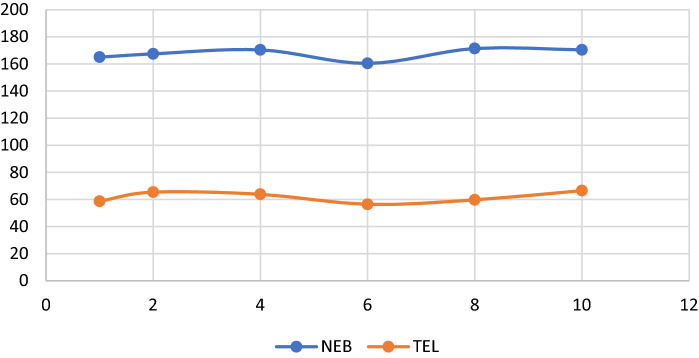
Effect of volume of buffer on the relative synchronous fluorescence intensity of (100.0 ng/ mL) for each NEB and TEL.

Moving to the next level different volumes of acetate buffer pH 4 were tested (1–10 ml), and there was no difference between different volumes. Consequently, a simpler, and environmentally benign buffer volume of 1 ml was used (Fig. 8).

#### Effect of organized media

**Figure 9:**
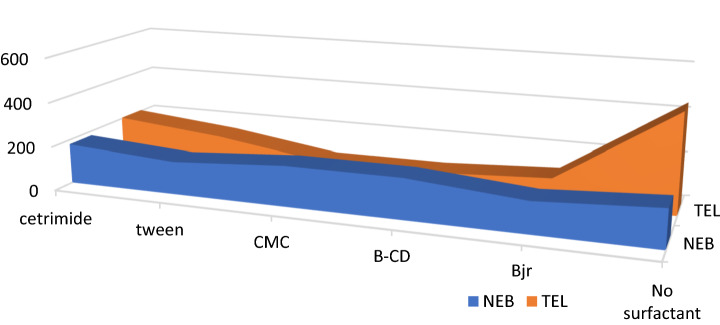
Effect of organized media on the relative synchronous fluorescence intensity of (100.0 ng/ mL) for each NEB and TEL.

Different types of organized media were studied including, SDS, cetrimide, tween- 80, β-CD, and CMC. None of them had any effect on the SF intensity of any of the two drugs (Fig. 9). The bulkiness and branched structure of the two compounds and the impossibility of being trapped in the cavities of the formed micelles can entail this observation.

### Method validation

The validation of the optimized fluorescence technique was performed for evaluation of method performance based on the ICH Q2R1 guidelines^44^.

#### Linearity and range

Method I: A linear correlation of the calibration graph based on SF intensity for TEL at 335 nm against the drug concentrations was established using the preselected experimental conditions. The linear concentration range was found to be (50–800 ng/mL for TEL) as abridged in Table 1.

$${\text{SFI}}_{{\uplambda 335\;{\text{mm}}}} = 1.203{\text{C}}_{{{\text{TEL}}}} - 35.07\;\left( {{\text{r}} = 0.9999} \right)$$

where: SFI is synchronous fluorescence intensity, C_TEL_ is TEL concentration in ng/mL.

**Table 1:** Analytical performance data for the determination of TEL alone and NEB/ TEL simultaneously by the proposed methods.

Parameter	Studied drugs		
Method mode	SF	D^1^	
Drug	TEL	NEB	TEL
Wavelength (nm)	335	296.3	320.5
Linearity range (ng/mL)	50.0–800.0	30.0–550.0	50.0–800.0
Intercept (*a*)	−35.072	2.340	−1.431
Slope (*b*)	1.203	0.110	0.055
Correlation coefficient (*r*)	0.9999	0.9999	0.9999
S.D. of residuals (S_*y/x*_)	5.490	0.366	0.153
S.D. of intercept (S_*a*_)	3.659	0.263	0.102
S.D. of slope (S_*b*_)	0.008	0.0008	0.0002
% RSD	0.704	0.875	0.752
*%* Error	0.266	0.330	0.284
DL(ng/ mL)	10.04	7.89	6.13
QL(ng/ mL)	30.41	23.92	18.58

Method II: The calibration graphs presented a straight line correlation based on the peak amplitude (D^1^) of the SFI value for NEB at 296.3 nm and TEL at 320.5 nm. The linear concentration ranges were found to be 30–550 ng/mL for NEB, and 50–800 ng/mL for TEL (Table 1).

$${\text{D}}_{{\uplambda 296.3\;{\text{mm}}}}^{1} = 0.11{\text{C}}_{{{\text{NEB}}}} + 2.34\;\left( {{\text{r}} = 0.9999} \right)$$

$${\text{D}}_{{\uplambda 320.5{\text{mm}}}}^{1} = 0.055{\text{C}}_{{{\text{TEL}}}} - 1.46\;\left( {{\text{r}} = 0.9999} \right)$$

where: D^1^ is the first derivative peak amplitude, and C is drug concentration in ng/mL.

The obtained statistical analysis data confirmed calibration graphs' linearity^45^ as illustrated in Table 2.

**Table 2:** Application of the proposed method for the determination of TEL alone and NEB/TEL simultaneously in raw materials.

Parameters	Studied drugs									Comparison methods	
	SFS			D^1^							
	TEL			NEB			TEL			NEB ^9^	TEL ^23^
	at (335 nm)			at (296.3 nm)			at (320.5 nm)				
	Taken conc. (ng/mL)	Found conc. (ng/mL)	% Found	Taken Conc. (ng/mL)	Found conc. (ng/mL)	% Found	Taken Conc. (ng/mL)	Found conc. (ng/mL)	% Found	% Found	% Found
	50.0	49.927	99.85	30.0	29.609	98.70	50.0	49.651	99.30	101.20	98.00
	100.0	100.625	100.62	100.0	99.545	99.55	100.0	100.560	100.56		
	200.0	200.774	100.39	200.0	201.271	100.64	200.0	202.378	101.19	99.59	100.81
	400.0	396.918	99.23	300.0	301.179	100.39	400.0	398.742	99.69		
	500.0	498.564	99.71	400.0	396.548	99.14	500.0	495.106	99.02	100.00	99.79
	700.0	708.171	101.17	500.0	505.539	101.11	700.0	698.742	99.82		
	800.0	795.023	99.38	550.0	546.956	99.45	800.0	802.379	100.30		
Mean(X¯)			100.05			99.85			99.98	100.26	99.53
± SD			0.700			0.870			0.750	0.837	1.422
%RSD			0.704			0.875			0.752	0.835	1.429
% Error			0.266			0.330			0.284	0.483	0.821
N	7			7			7				
t-test*	0.799(2.31) *			0.686(2.31) *			0.675(2.31) *				
F-test*	4.085(5.14) *			1.090(19.32) *			3.574(5.14) *				

N.B. Each result is the average of three separate determines.

*The values between parentheses are the tabulated t and F values at *P* = 0.05^45^.

#### DL and QL values

Detection and quantitation limits were calculated for both methods using the values of standard deviations of y-intercepts of regression lines and slopes^44^. The obtained results are summarized in Table 1.

#### Accuracy and precision

The developed techniques were applied for TEL raw material (alone) *via* synchronous spectrofluorimetry (Method I) in addition to NEB and TEL in-mixture simultaneously via the first derivative synchronous spectrofluorimetric method (Method II) over the established concentration ranges. Satisfactory % recoveries and low values of % RSD assured the good accuracy of the proposed methods, as shown in Table 2. To prove the accuracy of the proposed methods, the results of the assay of the studied drugs were compared with those obtained using reported methods^9,23^.

Data in Table 2 and Table 3 illustrate the accuracy and precision of the two methods, and they have no remarkable differences, as assured by statistical analysis of the data using the variance ratio F-test and the Student t-test.

**Table 3:** Precision data for NEB and TEL estimation using the studied synchronous and first derivative synchronous spectrofluorimetric techniques.

Parameters		SFS			D^1^					
		TEL (ng/mL)			NEB (ng/mL)			TEL (ng/mL)		
Conc		200	400	500	200	300	400	200	400	500
Intra- day	% Found	99.83	99.90	100.17	100.13	99.54	100.20	100.23	100.13	99.67
	± SD	± 0.666	± 0.346	± 0.551	± 0.598	± 0.561	± 0.401	± 0.642	± 0.416	± 0.751
	% RSD	0.667	0.347	0.550	0.597	0.564	0.400	0.641	0.416	0.753
	% Error	0.385	0.200	0.318	0.345	0.326	0.231	0.370	0.240	0.435
Inter- day	% Found	99.77	99.97	99.48	99.80	100.15	99.48	99.53	100.10	99.62
	± SD	± 0.551	± 0.777	± 0.660	± 0.625	± 0.304	± 0.660	± 0.651	± 0.369	± 0.534
	% RSD	0.552	0.777	0.663	0.626	0.304	0.663	0.654	0.368	0.536
	% Error	0.319	0.449	0.383	0.361	0.176	0.383	0.378	0.212	0.309

N. B. Each result is the average of three separate determinations.

#### Selectivity

The suggested techniques were successfully employed to estimate NEB and TEL without interference, both in the case of the synthetic mixtures and co-formulated dosage forms. The selectivity was assured using % error, as shown in Tables 4 and 5.

**Table 4:** Assay results for the concurrent assessment of TEL and NEB in synthetic mixtures using the studied synchronous and first derivative synchronous spectrofluorimetric techniques.

	^TEL and NEB^								
	SYN (for TEL)			D^1^ (ratio 1:8)					
	Taken conc. (ng/mL)	Found conc. (ng/mL)	% Found*	Taken conc. (ng/mL)	Taken conc. (ng/mL)	Found conc. (ng/mL)	Found conc. (ng/mL)	% Found*	
	TEL			NEB	TEL	NEB	TEL	NEB	TEL
1	320	318.08	99.40	40	320	39.76	321.28	99.40	100.4
2	480	481.44	100.30	60	480	60.42	478.08	100.70	99.60
3	560	561.12	100.20	70	560	70.63	565.04	100.90	100.90
Mean			99.97					100.33	100.30
± S.D			± 0.493					± 0.814	± 0.656
%RSD			0.493					0.811	0.654
% Error			0.285					0.468	0.378

*Mean of three determinations.

**Table 5:** Determination of TEL and NEB in dosage forms using the synchronous and first derivative synchronous spectrofluorimetric methods.

Parameters	Studied drugs									Comparison methods	
	SFS			D1							
	TEL			NEB			TEL			NEB^9^	TEL^23^
	at (335 nm)			at (296.3 nm)			at (320.5 nm)				
	Taken conc. (ng/mL)	Found conc. (ng/mL)	% Found	Taken conc. (ng/mL)	Found conc. (ng/mL)	%Found	Taken conc. (ng/mL)	Found conc. (ng/mL)	% Found	%Found	%Found
	400.0	402.80	100.70	50.0	49.45	98.90	400.0	401.20	100.30	100.64	98.00
	560.0	561.12	100.20	70.0	69.44	99.20	560.0	553.84	98.90	98.87	99.20
	800.0	792.00	99.00	100.0	100.10	100.10	800.0	797.60	99.70	100.14	101.09
Mean(X¯)			100.06			99.93			99.40	99.88	99.43
±SD			±0.792			±0.733			±0.624	±0.912	±1.558
%RSD			0.792			0.734			0.628	0.913	1.567
% Error			0.299			0.277			0.363	0.527	0.905
N	3			3			3				
t-test	0.520(2.776) *			0.757(2.776) *			0.206(2.776)*				
*F-test*	3.179(19.00) *			2.135(19.00) *			4.919(19.00)*				
*Nominal content*	40.024±0.792			4.997±0.733			39.760±0.624				

*N.B.* Each result is the average of three separate determines.

*The values between parentheses are the tabulated t and F values at *P* = 0.05^45^.

Thereafter, the technique selectivity was confirmed by determining NEB and TEL in spiked plasma without noticeable interference from plasma endogenous components. The small values of SD were obtained as presented in Table 6.

**Table 6:** Recovery data of TEL and NEB from spiked human plasma by the proposed synchronous fluorescence spectroscopy method (for TEL) and first derivative synchronous fluorescence spectroscopy method (for NEB and TEL).

	Method (1)		
	Added amount (ng/ mL)	Found amount (ng/ mL)	% Found
TEL SFS	80	81.52	101.90
	100	101.50	101.50
	150	153.47	102.31
Mean(X¯)	101.90		
± SD	± 0.405		
%RSD	0.397		
% Error	0.229		
	Method (2)		
	Added amount (ng/ mL)	Found amount (ng/ mL)	% Found
NEB D^1^	80	81.73	102.16
	100	101.20	101.20
	150	153.30	102.20
Mean(X¯)	101.85		
± SD	± 0.566		
%RSD	0.556		
% Error	0.321		
	Added amount (ng/ mL)	Found amount (ng/ mL)	% Found
TEL D^1^	80	80.82	101.02
	100	102.37	102.37
	150	154.05	102.70
Mean(X¯)	102.03		
± SD	± 0.89		
%RSD	0.872		
% Error	0.503		

### Applications

#### Synthetic mixtures analysis

SF and D^1^ SF methods were used to estimate NEB/TEL in the prepared synthetic mixtures. The accuracy of the suggested techniques was confirmed by the obtained results, as illustrated in Table 4.

#### Dosage forms analysis

The proposed technique was successfully used in TEL determination, either alone or in combination with NEB in their dosage forms. The results illustrated in Table 5 were acceptable compared to those obtained using reported techniques^9,23^. Statistical analysis revealed no remarkable variation in the accuracy and precision of the two techniques applying the variance ratio F-test and the Student's t-test^45^.

#### Biological applications

- **(a) Estimation of TEL in spiked human plasma (Method I)**

The high sensitivity of the developed method enabled the quantification of TEL in human plasma samples. Using previously optimized experimental parameters, the derived regression equation was utilized to analyze TEL in plasma, as shown in Table 6 with the following regression equation:

$${\text{y}}\, = \,1.44{\text{C}}_{{{\text{TEL}}}} - 14{\text{ at }}335 {\text{nm}}$$

The calibration curve for TEL determination was linear over the concentration range of (50.0–200.0 ng/ mL. The previously reported protein precipitation technique was adopted successfully, which is considered an add-on merit of the developed method^35^. The assay results of TEL in spiked human plasma samples were presented in Table 6. Satisfactory percentage recoveries were obtained (101.9 ± 0.41).

- (b) **Simultaneous estimation of NEB and TEL in human plasma (Method II)**

The sensitivity of those two methods was high enough to allow their determination. The proposed method was successfully used for the simultaneous determination of NEB and TEL in spiked human plasma based on the precipitation of protein. The linear calibration curves for NEB and TEL determination by the proposed D^1^SF methods were obtained over the concentration range of (50–200 ng/mL) for both NEB and TEL**.** The following regression equations in the plasma were obtained:

$${\text{y}} = 0.1093{\text{C}}_{{{\text{NEB}}}} + 1.75\;{\text{r}} = 0.9994\;\left( {\text{Method II}} \right)$$

$${\text{y}} = 0.464{\text{C}}_{{{\text{TEL}}}} + 0.05\;\;{\text{r}} = 0.9996\;\left( {{\text{Method}}\;{\text{II}}} \right)$$

with mean percentage recoveries of 101.85 ± 0.57and 102.03 ± 0.89 for NEB and TEL, respectively as represented in Table 6.

#### Fluorescence quantum yield of NEB

A fluorophore's quantum yield is an inherent property. Using a quantum yield reference, it is simple to determine the relative fluorescence quantum yield of fluorescent compounds. It is critical to select a fluorescence quantum yield reference that absorbs and emits in the same wavelength range as the investigated drug, whose quantum yield must be determined under specific measurement conditions (e.g., solvent/matrix, excitation wavelength, temperature, chromophore concentration). For the determination of Q of NEB, the single-point technique was utilized^46^, and phenol was used as the indicator, with a fluorescence quantum yield of 0.075 in hexane^47^. To avoid inner filter effects, the absorbance at λ_ex_ should be less than 0.1. To minimize experimental error, every sample absorbance and fluorescence spectra were calculated concurrently. NEB’s fluorescence quantum yield in methanol was found to be **0.22** using the corresponding Equation^46^:

$${\text{Q}}_{{\text{u}}} = {\text{Q}}_{{\text{R}}} \left[ {\frac{{{\text{I}}_{{\text{U}}} }}{{{\text{I}}_{{\text{R}}} }} \times \frac{{{\text{A}}_{{\text{R}}} }}{{{\text{A}}_{{\text{U}}} }}} \right] \cdot \frac{{{\text{n}}^{2} }}{{{\text{n}}^{2} }}$$

where: Q_R_, Q_u_: the FL quantum yield of phenol/hexane and NEB/methanol respectively. I_u_, I_R:_ NEB and phenol integrated fluorescence intensities. A_u_, A_R:_ NEB and phenol absorbance values. n: the solvent refractive index.

#### Assessment of the greenness of the developed techniques

Analysts play a key role in the environment and humans' protection from harmful organic wastes and solvents from pharmaceutical and chemical approaches. GAC is a domain that is regularly developed and updated. For analytical procedures 'environmental friendliness' assessment, numerous metrics can be followed for instance: Eco-scale and label can be assessed according to Tobiszewski M et al. ^37^. To evaluate the ecology of the method, an Eco-scale penalty paradigm is employed. The result of the analysis Eco-scale assessment is a penalty of deviation of 100 ("ideal green analysis"). The dots indicate the hazards used during the analysis. The greater the value, the more environmentally friendly the procedure is. Water is used instead of organic solvents in the proposed method. Furthermore, heating or energy-intensive processes that consume more than 0.1 kWh per sample were avoided. The proposed methods received an eco-scale score of **94**, indicating that they were excellent green methods (Table 7).

**Table 7:** Results for evaluation of greenness of the proposed approaches by the Analytical Eco-Scale score.

Reagents	Number of pictograms	Word sign	Penalty points
Acetate buffer pH 4 (1 ml)	1	Danger	2
Sodium acetate	1	Warning	1
Technique spectrofluorimetry			0
waste			3
Occupational hazard			0
Total penalty points			6

**Figure 10:**
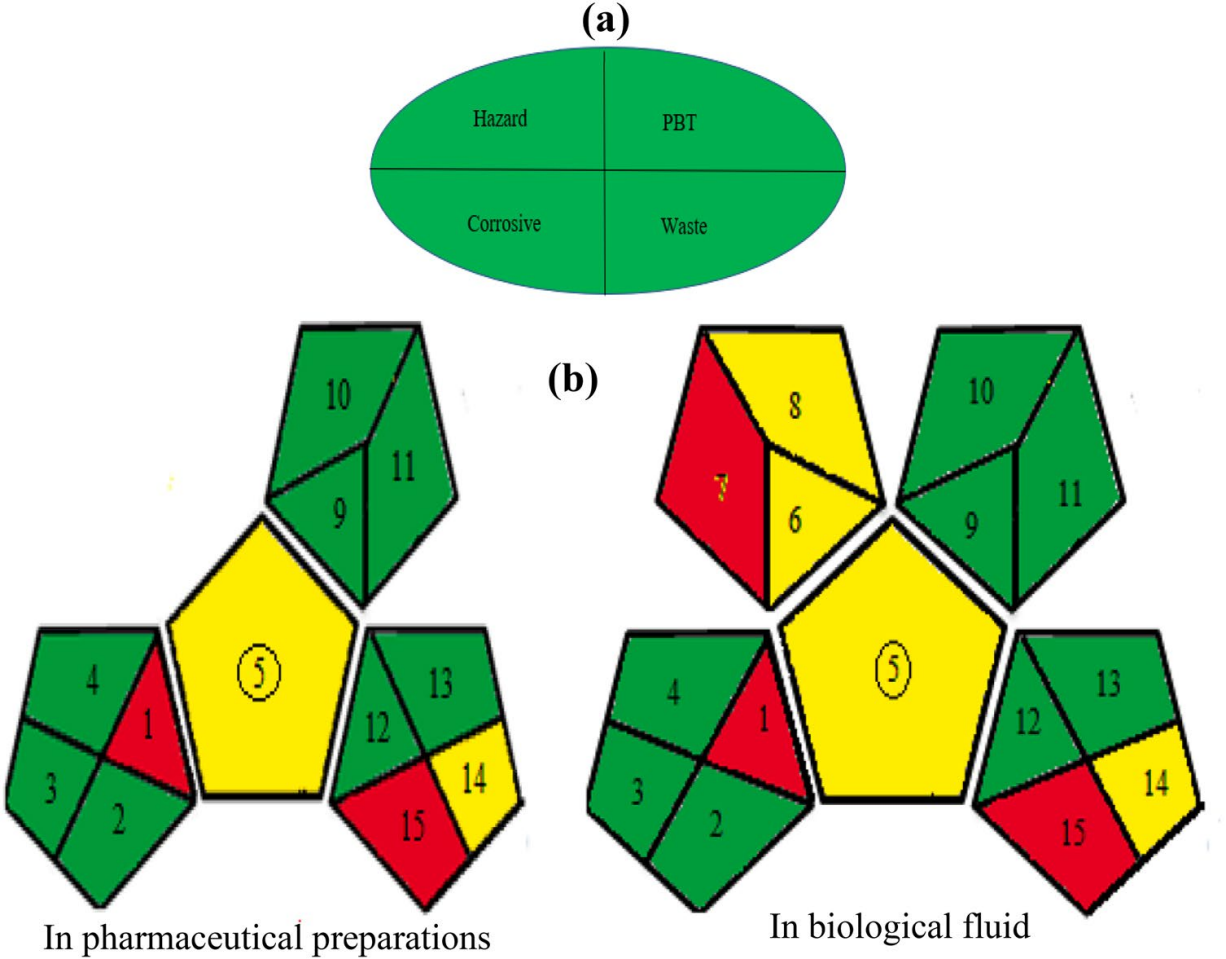
Evaluation of greenness of the proposed approaches by: (**a**) NEMI pictogram for both methods. (**b**) Green Analytical Procedure Index (GAPI) for each methods.

Another approach uses the NEMI, the oldest and easiest-to-read technique in the field, but has many drawbacks in practice, is time-consuming, and does not provide a quantitative measure of the effort involved to create its circular pictogram^37^. Briefly, this pictogram is divided into 4 sections, with each section colored white or green regulated by how well the relevant criteria are met. Four criteria are listed below: Except for the reagents used, the pH range should be in the range of 2 and 12; neither on the list of persistent, bioaccumulative, nor toxic chemicals^48^ nor on the list of hazardous wastes^49^. Finally, the net waste volume should not be more than 50 g or L. Based on the criteria listed above, our process meets all the requirements for certification as a green process (Fig. 10a).

A new tool has recently emerged: the GAPI^38^. It is considered one of the latest and greatest proposals for assessing environmental friendliness. This tool successfully overcomes the hurdles associated with the previously-mentioned tools. The GAPI tool uses pictograms for every stage of the analysis process greenness degree classification by a three-level color scale; green, yellow, or red. Through GAPI applying to the studied spectrofluorimetry: found to meet most GAPI standards. Boxes 1 and 15 are red due to being representative of off-line sampling and untreated waste, respectively. Boxes 5 and 14 are yellow due to simple procedure for sample preparation and 10 mL waste. In addition, GAPI has been applied to methods for drugs analysis in biological matrices; microextraction (yellow) was found to be simple, but acetonitrile using as a non-green solvent (red). A general conclusion of this visual representation is that our studied methods is broadly consistent with the green parameters of GAPI as they have minimal impact on human health and the environment (Fig. 10b).

## Conclusion

Rapid, selective, and sensitive green methods were developed to quantify TEL alone and in combination with NEB in co-formulated pharmaceutical preparations. The high sensitivity and selectivity of the method expanded its applications for the simultaneous determination of NEB and TEL in spiked human plasma without interference from endogenous components. The validated method is rapid and highly sensitive, thus can be used as an effective tool to monitor the therapeutic levels of the two drugs for in-patients.

## Data Availability

The datasets generated and/or analyzed during the current study are included in this published article.
